# Elicitor-Driven Defense Mechanisms: Shielding Cotton Plants against the Onslaught of *Cotton Leaf Curl Multan Virus* (CLCuMuV) Disease

**DOI:** 10.3390/metabo13111148

**Published:** 2023-11-12

**Authors:** Muhammad Fahad Khan, Ummad Ud Din Umar, Abdulwahed Fahad Alrefaei, Muhammad Junaid Rao

**Affiliations:** 1 Department of Plant Pathology, Faculty of Agricultural Sciences & Technology, Bahauddin Zakariya University, Multan 60800, Pakistan; mfkhan@gudgk.edu.pk; 2 Department of Plant Protection, Faculty of Agricultural Sciences, Ghazi University, Dera Ghazi Khan 32200, Pakistan; 3 Department of Zoology, College of Science, King Saud University, P.O. Box 2455, Riyadh 11451, Saudi Arabia; 4 College of Horticulture and Forestry, Huazhong Agricultural University, Wuhan 430070, China

**Keywords:** SAR, BTH, antioxidant activities, biotic stress, secondary metabolites, polyphenols

## Abstract

Salicylic acid (SA), benzothiadiazole (BTH), and methyl jasmonate (MeJA) are potential elicitors found in plants, playing a crucial role against various biotic and abiotic stresses. The systemic acquired resistance (SAR) mechanism was evaluated in cotton plants for the suppression of *Cotton leaf curl Multan Virus* (CLCuMuV) by the exogenous application of different elicitors. Seven different treatments of SA, MeJA, and BTH were applied exogenously at different concentrations and combinations. In response to elicitors treatment, enzymatic activities such as SOD, POD, CAT, PPO, PAL, β–1,3 glucanse, and chitinase as biochemical markers for resistance were determined from virus-inoculated and uninoculated cotton plants of susceptible and tolerant varieties, respectively. CLCuMuV was inoculated on cotton plants by whitefly (*Bemesia tabaci* biotype Asia II-1) and detected by PCR using specific primers for the coat protein region and the Cotton leaf curl betasatellite (CLCuMuBV)-associated component of CLCuMuV. The development of disease symptoms was observed and recorded on treated and control plants. The results revealed that BTH applied at a concentration of 1.1 mM appeared to be the most effective treatment for suppressing CLCuMuV disease in both varieties. The enzymatic activities in both varieties were not significantly different, and the disease was almost equally suppressed in BTH-treated cotton plants following virus inoculation. The beta satellite and coat protein regions of CLCuMuV were not detected by PCR in the cotton plants treated with BTH at either concentration. Among all elicitors, 1.1 mM BTH was proven to be the best option for inducing resistance after the onset of CLCuMuV infection and hence it could be part of the integrated disease management program against *Cotton leaf curl virus*.

## 1. Introduction

Cotton holds crucial significance for the textile sector, and Pakistan ranks as the fourth largest country in terms of harvested area and yield. The main hurdle in crop production is *Cotton leaf curl virus* (CLCuV) disease, belonging to the family Gemniviridae, of genus *Begomovirus,* which is transmitted in a persistent manner by whitefly (*Bemisia tabaci* Genn) [1,2,3,4]. This viral disease is known for causing substantial losses to cotton in Pakistan. CLCuV disease was observed for the first time in 1967 at Tibba Sultan Pur near District Multan, Pakistan [5]. This disease was consistently present in the cotton, but it did not cause significant losses to cotton until 1986. The disease was outspread in an epidemic form in 1991 and caused serious damage to the cotton by reducing its production and yield from 12.84 million bales to 8.05 million bales in 1994 [6]. From 2006 to 2012, a loss of 6.77 million bales amounting to 1.48 billion dollars was perceived to the national economy [7]. After the appearance of new resistance breaking the Burewala CLCuV, the production rate became slower, and considerable losses of 7.1 million bales amounting of 1.2 billion dollars were also observed in cotton [8]. The outbreak of CLCuV in Pakistan attracted worldwide attention, highlighting the urgency of addressing this significant disease [9,10].

Since then, considerable approaches have been deployed for the management of this disease, including cultural practices, control of insect vectors with insecticides, and the development of resistant cotton varieties by conventional breeding, host, and pathogen-derived resistance; it is pertinent to mention that this virulent strain appeared in Pakistan in 1994 and in 2006 in China [11,12]. However, the resistance was broken down due to the development of a new virulent strain of the virus which caused epidemics. The breeding of new resistant varieties is a time-consuming and temporary method due to viral recombination, which allows new strains to overcome resistance. Whitefly is the main vector responsible for the transmission of this disease and can be managed to some extent by insecticides, but the overuse of these insecticides leads to the development of resistance in whiteflies and even damages the ecosystem. Managing whiteflies proves challenging due to the wide range of host plants in which they can survive and multiply. Pesticides are an option but not a solution and have an impact on insect resistance and human and animal health. Considering the above-mentioned problems in managing CLCuV, we need to establish novel strategies [11]. Systemic acquired resistance (SAR) is now considered a management strategy for many diseases, but very little work has been conducted on the management of Geminiviruses in this regard. There is a need to exploit the SAR mechanism against *Begomovirus* in general and specifically against CLCuV. Therefore, an attempt was made to evaluate the SAR mechanism in cotton plants against CLCuV by the exogenous application of different SAR-inducer compounds which described SAR as a series of biochemical reactions that have a crucial role in plant defense against pathogens. The plant responds to salicylic acid (SA)-dependent signaling flow, which results in the systemic expression of a broad spectrum and long-lasting disease resistance that consequently produces pathogenesis-related (PR) proteins and phytoalexins against different pathogen infections. Salicylic acid (SA) is a signaling molecule that induces disease resistance responses both locally and systemically. Pathogens that invade plants can recognize and respond to their attack by activating their defense mechanism. This activation requires signal transmission from the receptor to the cell gene. Signaling molecules are involved in signal transmission in response to various pathogen attacks as well as in abiotic stresses [11]. Plants react quickly to an invasion of any pathogen by triggering a range of responses and induce resistance mechanisms that not only defend against acute diseases but also protect against potential infections. These responses are activated by the production of signaling hormones such as salicylic acid (SA), BTH and jasmonic acid (JA) on the onset of infection [12]. The expression of these elicitors activates the defense system of the whole plant, and this phenomenon is termed systemic acquired resistance (SAR), which offers a wide range of extensive and durable protection. SAR induction stimulates the plant for a more vigorous response to successive infections of various types of pathogens [13]. The application of SA enhances tolerance to stress, which depends on the developmental stage of plants. Salicylic acid is an endogenous regulator and plays a certain role in defense mechanisms against biotic and abiotic stresses [14].

Benzothiadiazole (BTH) can activate the SAR pathway downstream from SA signaling. As the outcome of the defense reaction, pathogenesis-related (PR) proteins, phenolic compounds, and enzymatic and non-enzymatic antioxidants are produced, which can suppress virus replication [15]. Antioxidant compounds are also produced at the same time, which scavenges the reactive oxygen species produced as a result of the interaction between plants and pathogens and protects them from oxidative damage. The proteins phenolic compounds catalase (CAT), phenylalanine ammonia-lyase (PAL), superoxide dismutase (SOD), peroxidase (POD), and polyphenol oxidase (PPO) could be used as biochemical markers for virus inhibition [16]. The stimulation of SOD can be a defensive response of the plant cell against the foyer of foreign organisms [17,18]. Benzothiadiazide (BTH) is a chemical analog of SA. It has been used effectively to induce resistance against an extensive variety of diseases in field crops. BTH is a powerful SAR activator downstream of SA signaling. It does not have antimicrobial properties. It increases crop resistance by activating the SAR in many plant species, such as beans, cauliflower, cucumber, tobacco, apple, and pea. BTH induces geminivirus resistance in pepper plants without damaging fruit quality and yield. Considering the importance of these enzymatic activities in plants, the objective of this study was to induce SAR in cotton against CLCuMuV by exogenous application of S. A, Methyl jasmonate (MeJA) (a derivative of jasmonic acid) and BTH at different concentrations and combinations under controlled conditions. Therefore, an attempt was made to enhance the resistance of the tolerant and susceptible varieties by the exogenous application of the elicitors. The resultant effect of defense-related enzymes, antioxidants, phenolics and PR proteins (e.g., β–,3-glucanase and chitinase) were quantified spectrophotometrically at different intervals. The main objective of this research was to evaluate the SAR mechanism in cotton plants against CLCuMuV, which ultimately would be helpful to develop another approach for the integrated management of CLCuMuV disease by evolving the best applicable strategy after the analysis of all experimental treatments.

## 2. Materials and Methods

### 2.1. Planting Material

All experiments were carried out in the glasshouse and laboratory of the Department of Plant Pathology Bahaudin Zakariya University, Multan (30.2705° N, 71.5024° E, 121 ft above from the sea level). Cottonseed varieties Cyto 124 (tolerant) and CIM-496 (highly susceptible) were collected from the Central Cotton Research Institute (CCRI), Multan, Pakistan, and grown in a glasshouse in earthen pots of 12 × 9 × 6 inches filled with a proportion of 1:3 of sandy loam soil with organic matter.

### 2.2. Application of Elicitors (Uninoculated)

To induce resistance in cotton, elicitors such as methyl jasmonate (MeJA) (Sigma Aldrich, Burlington, MA, USA), salicylic acid (SA) (Sigma Aldrich, Burlington, MA, USA), and benzothiadiazole (BTH) (Sigma Aldrich, Burlington, MA, USA) were applied exogenously at the four-leaf stage. Seven treatments of elicitors were applied with different concentrations, and each treatment was replicated three times under a completely randomized design. There was also a control in the experiment where only distilled water was sprayed. The treatment details were T0 = distilled water, T1 = 5 mM S. A, T2 = 10 mM S. A, T3 = 1 mM Me J after 1 day 5 mM SA, T4 = 1 mM MeJA after 1 day 10 mM SA, T5 = 1.1 mM BTH, T6 = 2.2 mM BTH, T7 = 1 mM Me J and after 1 day 5 mM SA + 1.1 mM BTH. After every 10 days of application, samples were collected after 48 h of application for the analysis of enzymatic activities.

### 2.3. Rearing of A-Viruliferous Whiteflies

The culture of A-viruliferous whiteflies (*Bemisia tabacci*) Asia II-1 was collected from the Department of Entomology, Bahauddin Zakariya University, Multan. Viruliferous whiteflies were collected from cotton plants infected with *Cotton leaf curl Multan beta virus* (CLCuMuV) and later confirmed with PCR. The Multan strain of CLCuMuV was inoculated on healthy cotton plants through collected viruliferous whiteflies. After inoculation, the whiteflies were killed by insecticidal spray. The biotype of whitefly was also confirmed by sequencing after PCR using primers MD10/MD12 from mitochondrial region MtCo-I. A-viruliferous whiteflies (Asia II-1) were reared on cotton plants infected with CLCuMuV in bug dorm (cages) of 75 × 75 × 115 cm with 150 nylon mesh. Once the acquisition period was completed, the healthy treated plants were subjected to viruliferous whiteflies in the cages for inoculation.

### 2.4. Application of Elicitors Followed by Virus Inoculation and Evaluation of Disease Severity

Another separate experiment was performed in which elicitor treatments as given above were applied exogenously followed by CLCuMuV inoculation through whiteflies reared on infected plants in the cages. After two days of application of elicitors, the plants were exposed to viruliferous whiteflies for 48 h. Virus-inoculated plants without any elicitor application were treated as controls. The second and third applications of elicitors were given at 10-day intervals. The samples were collected when the disease symptoms appeared on the inoculated control. Disease development was evaluated by using the disease rating scale as mentioned by the authors of the reference [1] and tested by PCR. The disease rating scale was used to evaluate the symptom severity on the leaves by applying the following formula:

Disease severity index=(sum of all disease rating scores)/(total number of plants)×100/(maximum scale)

### 2.5. DNA Extraction and Confirmation of CLCuMuV Amplification through Polymerase Chain Reaction (PCR)

DNA from cotton leaves was extracted by using the modified CTAB method [19]. Amplification of the coat protein region was performed with the pairs of universal primers AV-core (GCCHATRTAYAGRAAGCCMAGRAT) and Ac-cor (GGRTTDGARGCATGHGTACANGCC) with an amplicon size of 579 bp [20]. The PCR conditions were as follows: initial denaturation at 95 °C for 1 min followed by 35 cycles of denaturation at 94 °C for 45 s, annealing at 58 °C for 45 s, and extension at 72 °C for 45 s, and a final step at 72 °C for 10 min. Amplification of the beta satellite was performed by the primer pair CLCuMuB-F11 GGTCCCACTGCTTGTCTTGA and CLCuMuB-F33 GGTTCATAGTCGACGTTCGC with an amplicon size of 481 bp [21] with the following PCR conditions: 95 °C for 3 min, 35 cycles of 95 °C for 1 min, annealing at 52 °C for 1 min, extension at 72 °C for 1 min and a final extension at 72 °C for 10 min. PCR was carried out in 25 µL reactions containing standard PCR ingredients and 1 µL of DNA as a template. PCR products were analyzed on 2% agarose gel.

### 2.6. Determination of Enzymatic Activities in Order to Observe Biological Changes in Plants

As a result of applications of elicitors on both uninoculated and virus-inoculated cotton plants, the resistance induced was evaluated by biochemical markers. Leaf samples were collected every 10 days after the exogenous application of elicitors to determine enzymatic and non-enzymatic antioxidant, catalase (CAT), peroxidase (POD), phenylalanine ammonia (PAL), superoxide dismutase (POD), and polyphenol oxidase (PPO) activities spectrophotometrically. Further pathogenesis-related proteins, such as β-1-3 glucanase and chitinase activities, were also determined.

### 2.7. Enzyme Extract for SOD, POD, and CAT

The activities of SOD, POD, and CAT in cotton leaves were examined according to the technique developed by [22]. Leaf samples of 0.2 g from each treatment were crushed in liquid nitrogen with a mortar and pestle, and 2 mL of 50 mM chilled phosphate buffer (pH 7.8) containing 1 mM ethylene diamine tetraacetic acid (EDTA) was added instantly. The homogenate was further crushed and then centrifuged at 10,000× *g* for 15 min at 4 °C. The upper phase was used to determine the enzymatic activity of CAT, SOD, and POD assays.

### 2.8. Assay for SOD (EC 1.15.1.1)

SOD activity was calculated based on its efficiency in preventing nitroblue tetrazole (NBT) photoreduction. The total volume of the reaction mixture was 3 mL, containing 0.1 mM EDTA, 130 mM methionine, 0.1 mL enzyme extract, 0.75 mM NBT, 50 mM phosphate buffer pH 7.8 and freshly prepared 0.02 mM riboflavin. The solution was exposed to a fluorescent 40-watt lamp for 10 min. Illuminated and non-illuminated reactions both with and without enzyme extracts were used as reference standards. The absorbance values of the blank control and the reaction mixture were measured at 560 nm with a spectrophotometer (UV Vis 3000, Bio-Rad Inc. Hercules, CA, USA). The quantity of an enzyme that was needed for 50 percent inhibition of the NBT photoreduction is considered equal to one unit of the SOD activity (U) and expressed in protein per Umg^−1^. The SOD activity was determined by using the following formula:

$$\begin{array}{cc} SOD\;activity= & \left(Abs.\;of\;blank-Abs.\;of\;tested\;sample\right)÷\left(Abs.\;of\;blank\right)\\ & ÷50\%\times \left(Total\;quantity\;of\;reaction\right)\\ & ÷(Conc.\;of\;protein\;in\;enzyme\;extract) \end{array}$$

### 2.9. Assay for POD (EC 1.11.1.7)

POD activity was determined by measuring the increase in the absorbance at 470 nm due to the formation of tetraguaiacol, an oxidation product of guaiacol [23]. The final concentration of the reaction mixture contained 33 mM potassium phosphate buffer (pH 6.1), 16 mM guaiacol, 2 mM H_2_O_2_, and 200 µL of enzyme extract. The increase in absorbance at 470 nm was monitored for 3 min with and without the addition of enzyme extract. One unit of POD was defined as the amount of enzyme that consumed 1 µmol of H_2_O_2_ min^−1^. POD activity (µmol H_2_O_2_ decomposed min^−1^ mg^−1^ protein or Umg^−1^ of protein) was calculated using the extinction coefficient of 26.6 mM^−1^ cm^−1^.

$$POD\;activity=\frac{Total\;volume\;in\;cuvette\times ∆A470\;{min}^{-1}}{The\;Volume\;of\;enzyme\;extract\times Protein\;conc.\times \;26.6\;M^{-1}\;{cm}^{-1}}$$

### 2.10. Assay for CAT (EC 1.11.1.6)

Catalase activity was assayed by measuring the rate of decomposition of H_2_O_2_ using the method of [24]. The reaction mixture consisted of 50 mM potassium phosphate buffer (pH 7.0), 12 mM H_2_O_2_ and 50 µL of enzyme extract. The rate of disappearance of H_2_O_2_ was followed by observing the rate of decrease in the absorbance at 240 nm for 3 min. One unit of CAT is defined as the amount of enzyme that decomposes 1 µmol H_2_O_2_ min^−1^ at 25 °C. An extinction coefficient of 43.6 mM^−1^ cm^−1^ M^−1^ cm^−1^ was used to calculate the CAT activity (µmol min^−1^ mg^−1^ protein or Umg^−1^ of protein) by using the formula below.

$$CAT\;activity=\frac{Total\;volume\;in\;cuvette\times ∆A240\;{min}^{-1}}{The\;Volume\;of\;enzyme\;extract\times Protein\;conc.\times \;43.6\;M^{-1}\;{cm}^{-1}}$$

### 2.11. Enzyme Extract and Assay for PAL (EC.4.3.1.24)

For the estimation of PAL activity, leaf samples were crushed in liquid nitrogen and further homogenized in 3 mL of ice-cold 0.1 M sodium borate buffer, pH 7.0, containing 1.4 mM 2-mercaptoethanol and 0.1 g of insoluble polyvinyl pyrrolidone. The extract was filtered through cheese cloth, and the filtrate was centrifuged at 12,000× *g* for 15 min. The supernatant was used as an enzyme source. PAL activity was determined as the rate of conversion of L-phenylalanine to trans-cinnamic acid at 290 nm as described by [25]. Samples containing 0.4 mL of enzyme extract were incubated with 0.5 mL of 0.1 M borate buffer, pH 8.8, and 0.5 mL of 12 mM L-phenylalanine prepared in the same buffer followed by incubation for 30 min at 30 °C. The amount of trans-cinnamic acid synthesized was calculated using its extinction coefficient of 9630 M^−1^ cm^−1^. Enzyme activity was expressed as nmol trans-cinnamic acid min^−1^ mg^−1^ protein or Umg^−1^ protein. The given formula was used to calculate PAL activity.

$$PAL\;activity=\frac{Total\;volume\;in\;cuvette\times ∆A290\;{min}^{-1}}{The\;Volume\;of\;enzyme\;extract\times Protein\;conc.\times \;9630\;M^{-1}\;{cm}^{-1}}$$

### 2.12. Enzyme Extract and Assay for PPO (EC. 1.14.18.1)

PPO activity was determined per the procedure given by [26]. Leaf samples (1 g) were thoroughly crushed in a mortar and pestle in the presence of liquid nitrogen and 2 mL of 0.1 M sodium phosphate buffer (pH 6.5) was added immediately after crushing for further homogenization. The extract was poured into a 1.5 mL Eppendorf tube and centrifuged at 12,000× *g* for 15 min at 4 °C. The obtained supernatant was used as the enzyme source. The reaction mixture consisted of 200 µL of the enzyme extract and 1.5 mL of 0.1 M sodium phosphate buffer (pH 6.5). To start the reaction, 200 µL of 0.01 M catechol was added, and the activity was expressed as the change in the absorbance at 495 nm min^−1^ mg^−1^ protein or U mg^−1^ protein. The enzymatic activity of PPO was calculated by putting the value of the extinction coefficient of 9630 mM^−1^ cm^−1^ in the formula below.

$$PPO\;activity=\frac{Total\;volume\;in\;cuvette\times ∆A495\;{min}^{-1}}{The\;Volume\;of\;enzyme\;extract\times Protein\;conc.\times \;3450\;M^{-1}\;{cm}^{-1}}$$

### 2.13. Enzyme Extraction for β–1,3-Glucanase and Chitinase and Colloidal Chitin for Chitinase Activity

Freshly detached 1 g cotton leaves from treated plants were homogenized using liquid nitrogen in precooled mortar and pestle vigorously followed by the addition of 2 mL of chilled 0.05 M Na-acetate buffer (pH 5.0) and transferred to a 1.5 mL centrifuge tube for centrifugation at 12,000× *g* for 15 min at 4 °C. The upper phase was used directly as an enzyme. The method defined by [27] was used to prepare colloidal chitin. In 175 mL of cold concentrated HCl, 10 g of crab-shell Chitin (Sigma-Aldrich, Burlington, MA, USA) was dissolved, and the mixture was rapidly stirred at 4 °C for 24 h. The mixture was poured into 1 L of cold ethanol and incubated for 24–48 h at −20 °C. The resulting suspension of chitin was passed through Whatman filter paper no. 1. To neutralize the pH of the mixture, water was added until it became neutral. The chitin was dried overnight at 90 °C and crushed into powder in a mortar and pestle.

### 2.14. Microplate Assay for β–1,3-Glucanase and Chitinase

Enzymatic production of chitinase and β–1,3-glucanase was estimated separately using 0.25% colloidal chitin and laminarin (Sigma-Aldrich, Burlington, MA, USA), respectively, as substrates [28]. The reaction was prepared by mixing 20 μL of each substrate solution with 10 μL of enzyme extract. The mixture was added to a 96-well non-skirted PCR plate (Bio-Rad Laboratories, Inc., Hercules, CA, USA) and sealed with Axygen^®^ sealing film (Corning Life Sciences, New York, NY, USA). The PCR plate was incubated for 15 min at 40 °C in a 96-well My Cycler (Bio-Rad Laboratories, Inc., Hercules, CA, USA). After incubation, 1.3% phenol (Sigma-Aldrich), 20% Na-K-tartrate, 100 μL of DNS reagent (Sigma-Aldrich) and 1.25% NaOH were mixed and heated for 5 min at 95 °C. The prepared mixture was cooled at room temperature, and 100 μL was added to a 96-well microtiter ELISA plate. The absorbance of reduced sugars in the microplate was calculated at 550 nm using an ELx800 ELISA reader (BioTek Instruments, Inc., Winooski, VT, USA). Enzymatic activity was calculated by the quantity of enzyme that produced μg of reducing sugar per min per mg of protein or Umg^−1^ under both sets of conditions.

### 2.15. Estimation of Total Phenolic Contents (EC 1.14.13.7)

Leaf samples (0.5 g) were homogenized in 2 mL of 80% methanol and agitated for 15 min at 70 °C [29]. One milliliter of the methanolic extract was added to 5 mL of distilled water and 250 µL of 1 N Folin-Ciocalteu reagent (Sigma-Aldrich, Burlington, MA, USA), and the solution was kept at 25 °C. The absorbance of the developed blue color was measured using a spectrophotometer at 725 nm. Gallic acid was used as the standard. The amount of phenolics was expressed as µg gallic acid mg^−1^ protein.

### 2.16. Bradford Protein Assay

For protein determination, leaves were extracted separately in sodium phosphate buffer (pH 7.2). Total proteins were estimated by the Bradford reagent, and bovine serum albumin was used as the standard [30].

### 2.17. Correlation, Principal Component and Statistical Analysis

The collected data was subjected to ANOVA and LSD analysis using statistical software package (SAS, 2002, Cary, NC, USA). The graphical representation of figures, principal component analysis and correlation matrix were developed by analyzing datasets by Origin software (2023) version 10.5.123. The correlation and principal component analysis of the datasets has been described in the results section with figures.

## 3. Results

The systemic acquired resistance (SAR) mechanism in the cotton plant was studied by inducing resistance through the exogenous application of elicitors. The induced resistance in cotton was evaluated by different enzymatic and antioxidant activities. In response to elicitor applications, resistance was induced in the cotton plant, and CLCuMuV was significantly suppressed as compared to the control.

### 3.1. Impact of Elicitors Treatments on Disease Severity of Cotton Leaf Curl Virus

**Figure 1:**
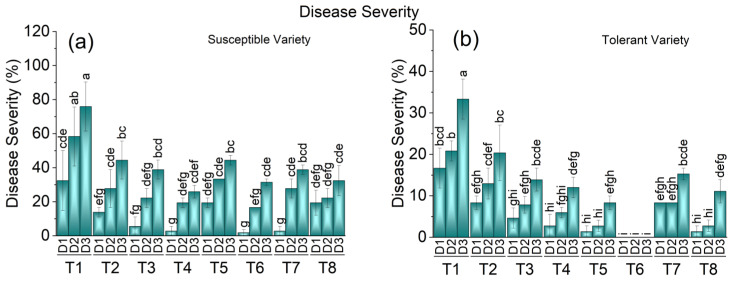
Mean disease severity of CLCuMuBV in susceptible (**a**) and tolerant varieties (**b**) on three dates in response to treatments; T1: control, T2: SA 5 mM, T3: SA10 mM, T4: MeJA1 mM + SA5 mM, T5: MeJA1 mM + SA10 mM, T6: 1.1 mM BTH, T7: 2.2 mM BTH, T8: 1 mM MeJA + 5 mM SA + 1.1 mM BTH. Different letters on bars show significant differences at *p* < 0.05.

The response of elicitor treatments on cotton plants against Cotton Leaf Curl Virus (CLCuV), disease severity was calculated based on visual symptoms in tolerant and susceptible varieties. The effects of all the treatments were significantly (*p* < 0.05) different in both the tolerant and susceptible varieties. In the tolerant variety, the disease was suppressed in all the treatments compared to the control. Maximum disease severity (24%) was recorded on the plants that were only subjected to virus inoculation without any application of elicitor treatments. The disease was completely suppressed when 1.1 mM BTH was applied, whereas only 1.23% disease severity was calculated for the plants treated with 2.2 mM BTH. The disease severity remained very low, at 2.76%, when all the treatments were applied in combination. In the susceptible variety, there was a substantial effect of all the treatments on disease severity compared to the control. In other treatments, the disease severity was significantly suppressed compared to the control. In the susceptible variety, the maximum disease severity (55%) was recorded on the control plants that were exposed to the virus only. Like the tolerant variety, BTH also responded well regarding the suppression of disease, where BTH applied at concentrations of 1.1 and 2.2 mM resulted in disease severities of 3.39% and 3.53%, respectively. There was a significant (*p* < 0.05) effect of all the different treatments on disease severity compared to the control. The exogenous application of BTH at concentrations of 1.1 mM and 2.2 mM on the susceptible variety appeared to be statistically significant and resulted in a significant effect on the disease severity. However, in the tolerant variety, no disease symptoms appeared when BTH was applied at 1.1 mM. Negligible disease severity was observed on the cotton plants treated with 2.2 mM BTH alone and in a combination of MeJA 1 mM+ S. A 5 mM + 150 mg/L BTH (Figure 1).

### 3.2. Confirmation of CLCuMuV Infection through PCR

**Figure 2:**
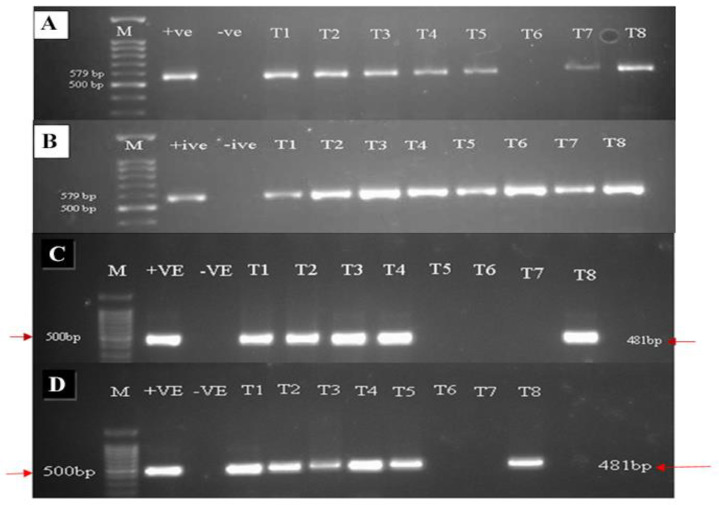
(**A**,**B**) One (1)% Agarose gel electrophoresis depicting the amplifications of coat protein genes in different cotton plants using AV/AC core primers. A: tolerant, B: susceptible. M: Lane M 100 bp ladder; Lane 1: positive control, Lane 2: negative control, Lanes 3–8. (**C**,**D**) 1% Agarose gel electrophoresis depicting the amplifications of CLCuMuB in different cotton plants using specific primers A: susceptible B: tolerant. M: Lane M 100 bp Marker, Lane 1: positive control, Lane 2: negative control: Lanes 3–8 treatments. T1: control, T2: SA 5 mM, T3: SA10 mM, T4: MeJA1 mM + SA5 mM, T5: MeJA1 mM + SA10 mM, T6: 1.1 mM BTH, T7: 2.2 mM BTH, T8: MeJA1 mM + SA 5 mM + 1.1 mM BTH.

Specific primers for the AV/AC core were used to target the coat protein gene region of Begomovirus (CLCuMuV). The extracted DNA of all the treated plants was subjected to PCR detection of CLCuV by amplification of AV/AC core primers. The virus was detected in all the treated cotton plants of the tolerant variety except BTH, which was exogenously applied at a concentration of 1.1 mM. In contrast, in the susceptible variety, CLCuMuV was detected and amplified by AV/AC core primers from extracted DNA of all the treated plants. Positive amplification of 579 bp is shown against each treatment. For the detection of Cotton leaf curl Multan betasatellite (CLCuMuB) another set of specific primers was used that amplified a 481 bp region out of 1.4 kb of CLCuMuB from the extracted DNA of treated plants. In the tolerant cotton variety, the beta component of CLCuMuV was detected in control plants where the only virus was inoculated and in other elicitor-treated cotton plants except where plants were treated with MeJA1 mM+ 10 mM SA and 1.1 and 2.2 mM BTH in the tolerant variety. In the susceptible variety, CLCuMuBV was not amplified only when BTH was applied at both concentrations (Figure 2A–D).

### 3.3. Impact of Elicitors Treatments on Defense-Related Enzymatic Activity of Cotton Plants

#### 3.3.1. Superoxide Dismutase (SOD) Enzymatic Activity

**Figure 3:**
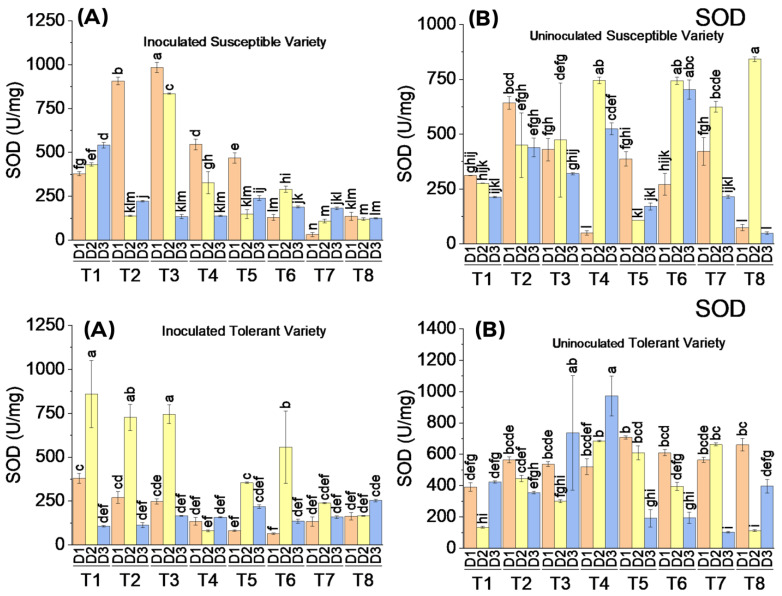
Mean values of SOD activity in susceptible ((**above A**) inoculated and (**above B**) uninoculated) and tolerant ((**below A**) inoculated and (**below B**) uninoculated) varieties on three dates in response to treatments; T1: control, T2: SA 5 mM, T3: SA10 mM, T4: MeJA1 mM + SA5 mM, T5: MeJA1 mM + SA10 mM, T6: 1.1 mM BTH, T7: 2.2 mM BTH, T8: 1 mM MeJA + 5 mM SA + 1.1 mM BTH. Different letters on bars show significant differences at *p* < 0.05.

SOD activity in tolerant and susceptible varieties of cotton treated with different elicitors showed varying responses. There was a significant difference in SOD activity between virus-inoculated and uninoculated cotton plants of the susceptible and tolerant varieties. SOD activity was not increased substantially in uninoculated plants treated only with elicitors. However, the enzymatic activity of SOD in virus-inoculated plants treated with elicitors showed an increasing trend on the second and third dates of analysis in both susceptible and tolerant varieties. A synergetic effect of virus infection followed by elicitor application was observed in the form of increased SOD activity. The SOD activity was increased in the controls of both varieties of virus-inoculated plants compared to the controls of uninoculated plants. The average maximum activity of SOD was observed in both tolerant and susceptible varieties where 10 mM SA was applied, whereas in the uninoculated plants, the impact of treatment 1 mM MeJA along with 5 mM SA, appeared to be dominant for the average SOD activity. The results revealed that virus inoculation followed by the application of elicitors had a remarkable effect on SOD activity (Figure 3).

#### 3.3.2. Peroxidase (POD) Enzymatic Activity

**Figure 4:**
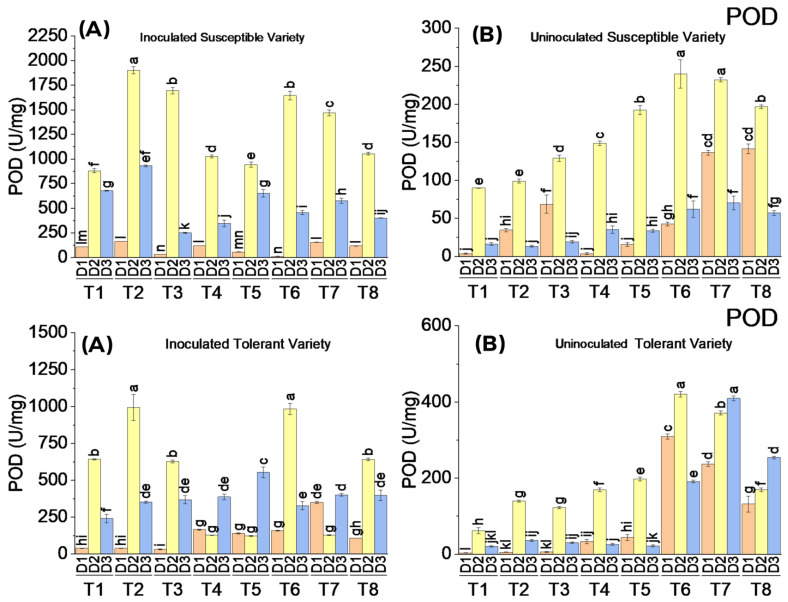
Mean values of POD activity in susceptible ((**above A**) inoculated and (**above B**) uninoculated) and tolerant ((**below A**) inoculated and (**below B**) uninoculated) varieties on three dates in response to treatments; T1: control, T2: SA 5 mM, T3: SA10 mM, T4: MeJA1 mM + SA5 mM, T5: MeJA1 mM + SA10 mM, T6: 1.1 mM BTH, T7: 2.2 mM BTH, T8: 1 mM MeJA + 5 mM SA + 1.1 mM BTH. Different letters on bars show significant differences at *p* < 0.05.

The application of elicitors did not affect POD activity in uninoculated cotton plants compared to virus-inoculated plants of both varieties. A maximum increase in POD activity of up to only 400 U/mg was observed in uninoculated plants, whereas there was a substantial increase in POD activity (up to 1900 U/mg) in virus-inoculated cotton plants of both susceptible and tolerant varieties compared to the control. The application of elicitors after virus inoculation tremendously increased the POD activity in both varieties on the second date of analysis compared to the control where the only virus was inoculated. On the third date of analysis, the POD activity was decreased. In the susceptible variety, more POD activity was observed than in the tolerant variety. The most effective treatments were T2 (5 mM SA) and T6 (1.1 mM BTH) regarding the POD activity in inoculated susceptible and tolerant varieties, respectively, on the second date of analysis. It is clear from the results that an increased level of POD activity was observed when the cotton plants were subjected to viral inoculation followed by the application of elicitors (Figure 4).

#### 3.3.3. Catalase (CAT) Enzymatic Activity

**Figure 5:**
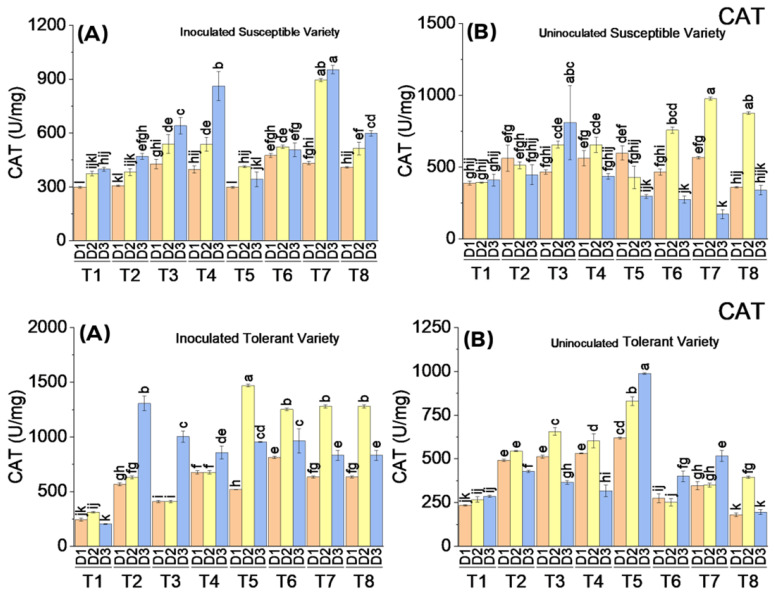
Mean values of CAT activity in susceptible ((**above A**) inoculated and (**above B**) uninoculated) and tolerant ((**below A**) inoculated and (**below B**) uninoculated) varieties on three dates in response to treatments; T1: control, T2: SA 5 mM, T3: SA10 mM, T4: MeJA1 mM + SA5 mM, T5: MeJA1 mM + SA10 mM, T6: 1.1 mM BTH, T7: 2.2 mM BTH, T8: 1 mM MeJA + 5 mM SA + 1.1 mM BTH. Different letters on bars show significant differences at *p* < 0.05.

Catalase (CAT) showed varying degrees of responses to the elicitor treatments in the susceptible and tolerant varieties. The enzymatic activity of CAT was significantly higher in the tolerant and susceptible cotton varieties in response to the treatments applied after virus inoculation. The maximum CAT activity was observed in virus-inoculated plants, followed by the application of treatments where BTH was applied at concentrations of 2.2 mM and 1.1 mM to susceptible and tolerant varieties, respectively, where BTH was applied at a concentration of 2.2 mM. The CAT activity increased in response to most of the treatments on the second date of analysis in the tolerant variety, whereas it increased on the third date of analysis in the susceptible variety. Overall, CAT activity tended to increase more after the application of elicitors in virus-inoculated cotton plants (Figure 5).

#### 3.3.4. Polyphenoloxidase (PPO) Enzymatic Activity

**Figure 6:**
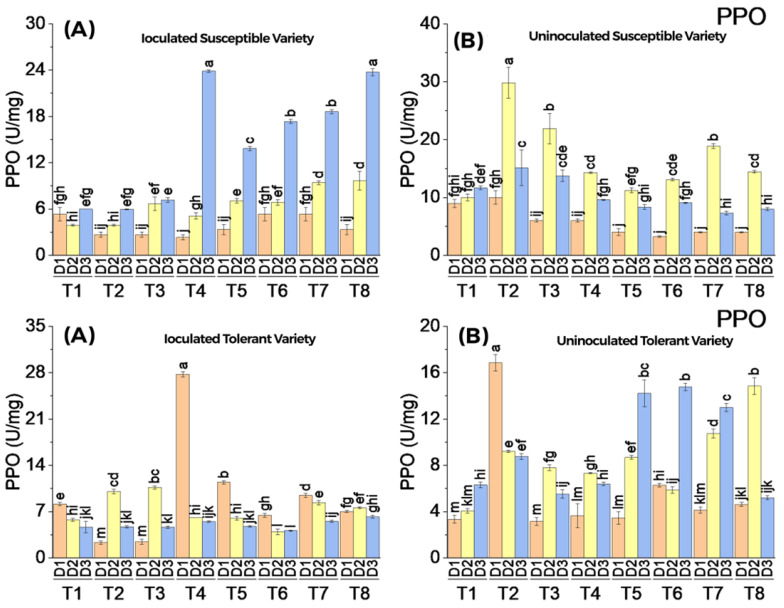
Mean values of PPO activity in susceptible ((**above A**) inoculated and (**above B**) uninoculated) and tolerant ((**below A**) inoculated and (**below B**) uninoculated) varieties on three dates in response to treatments; T1: control, T2: SA 5 mM, T3: SA10 mM, T4: MeJA1 mM + SA5 mM, T5: MeJA1 mM + SA10 mM, T6: 1.1 mM BTH, T7: 2.2 mM BTH, T8: 1 mM MeJA + 5 mM SA + 1.1 mM BTH. Different letters on bars show significant differences at *p* < 0.05.

Polyphenoloxidase (PPO) activity in both experiments showed a mixed response to the application of elicitors with and without virus inoculation. There was significant variability in PPO activity between uninoculated and virus-inoculated plants of the susceptible variety. Increased PPO activity on the third date of the analysis showed a synergistic effect of elicitors in virus-inoculated plants in treatments T4, T5, T6, T7, and T8. An increasing trend of PPO activity was observed on the second date of analysis in the uninoculated susceptible variety. Compared to the susceptible variety, the response of the tolerant variety to PPO activity appeared to be low and did not follow any increasing or decreasing trend with respect to the dates of analysis (Figure 6).

#### 3.3.5. Phenylalanine Amonialyase (PAL) Enzymatic Activity

**Figure 7:**
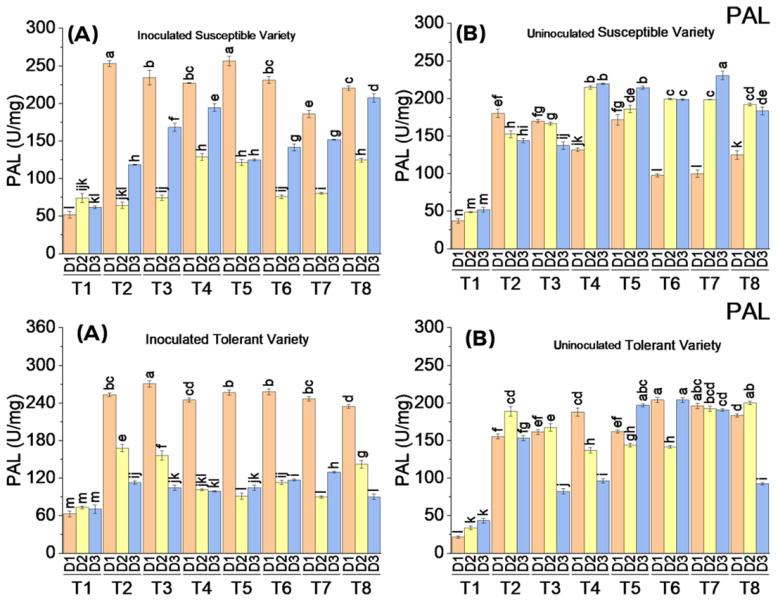
Mean values of PAL activity in susceptible ((**above A**) inoculated and (**above B**) uninoculated) and tolerant ((**below A**) inoculated and (**below B**) uninoculated) varieties on three dates in response to treatments; T1: control, T2: SA 5 mM, T3: SA10 mM, T4: MeJA1 mM + SA5 mM, T5: MeJA1 mM + SA10 mM, T6: 1.1 mM BTH, T7: 2.2 mM BTH, T8: 1 mM MeJA + 5 mM SA + 1.1 mM BTH. Different letters on bars show significant differences at *p* < 0.05.

Exogenous application of elicitors increased the PAL activity in all the treatments compared to the control in inoculated and uninoculated plants of both varieties. PAL activity was significantly higher in virus-inoculated plants of both varieties on the first date of analysis. Its activity was observed to be slightly higher in the tolerant variety than in the susceptible variety. However, PAL activity increased almost equally to the results of treatments on uninoculated plants of both varieties on the second and third dates of analysis. The PAL activity in uninoculated elicitors treated plants of susceptible and tolerant varieties increased up to 232 U/mg and 204 U/mg, respectively. However, it increased up to 256 U/mg and 271 U/mg in virus-inoculated treated plants of susceptible and tolerant varieties, respectively (Figure 7).

#### 3.3.6. Phenolic Activity

**Figure 8:**
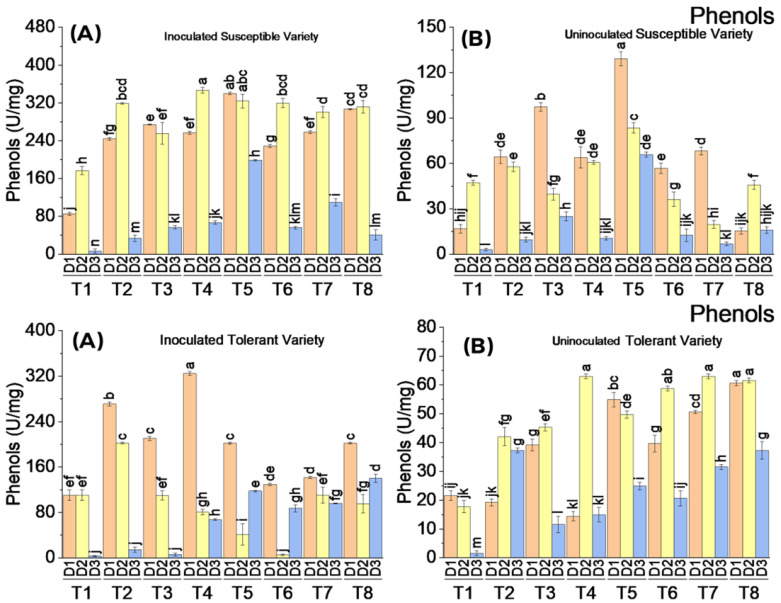
Mean values of phenolic activity in susceptible ((**above A**) inoculated and (**above B**) uninoculated) and tolerant ((**below A**) inoculated and (**below B**) uninoculated) varieties on three dates in response to treatments; T1: control, T2: SA 5 mM, T3: SA10 mM, T4: MeJA1 mM + SA5 mM, T5: MeJA1 mM + SA10 mM, T6: 1.1 mM BTH, T7: 2.2 mM BTH, T8: 1 mM MeJA + 5 mM SA + 1.1 mM BTH. Different letters on bars show significant differences at *p* < 0.05.

There was a significant difference in phenolic compounds produced in virus-inoculated and uninoculated plants of both tolerant and susceptible varieties. Virus inoculation followed by the application of elicitors showed a prominent increase in the total phenolic contents of the susceptible variety on the first and second dates of the analysis. All the treatments showed a significant increase in phenolic activity compared to the control on the first and second dates of analysis, whereas it was decreased on the third date of analysis. The phenolic compounds were produced in almost equal amounts as the result of all the treatments on the first and second dates of analysis. In uninoculated plants, there was a very low quantity of phenolic compounds produced as a result of the application of elicitors. In the tolerant variety, there was a substantial increase in the phenolic compounds as the result of virus inoculation followed by the application of elicitors only on the first date of analysis. The maximum increase in phenolic compounds was recorded to be 325 U/mg on the first date of analysis where SA was applied at a concentration of 10 mM. However, a very low quantity of phenols was recorded when the plants were treated with the exogenous application of elicitors without virus inoculation (Figure 8).

#### 3.3.7. Beta 1,3 Glucanase Activity

**Figure 9:**
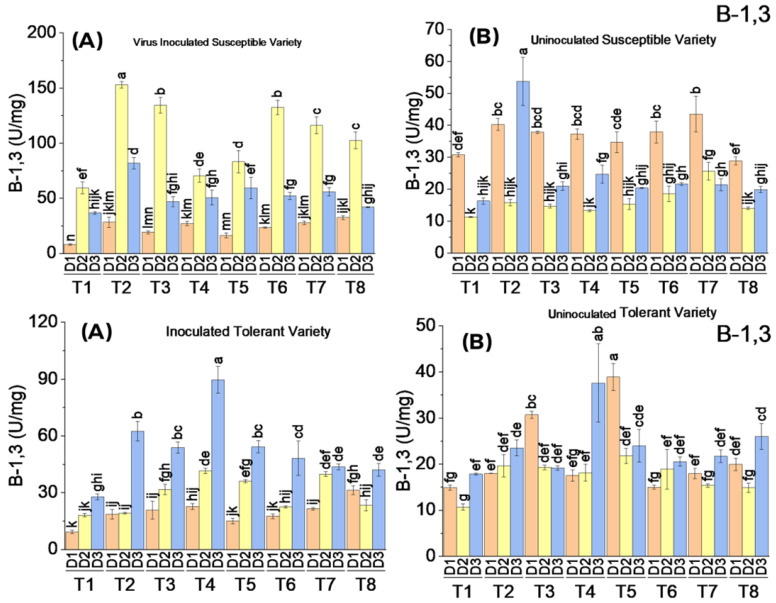
Mean values of Beta 1,3 glucanase activity in susceptible ((**above A**) inoculated and (**above B**) uninoculated) and tolerant ((**below A**) inoculated and (**below B**) uninoculated) varieties on three dates in response to treatments; T1: control, T2: SA 5 mM, T3: SA10 mM, T4: MeJA1 mM + SA5 mM, T5: MeJA1 mM + SA10 mM, T6: 1.1 mM BTH, T7: 2.2 mM BTH, T8: 1 mM MeJA + 5 mM SA + 1.1 mM BTH. Different letters on bars show significant differences at *p* < 0.05.

The application of elicitor treatments without virus inoculation did not significantly affect β–1,3-glucanase activity either in the tolerant or susceptible cotton variety. The activity in all treated cotton plants of both varieties remained very low. It increased only up to 42 U/mg in plants treated with BTH @ 2.2 mM, whereas in the control, it was only 37 U/mg. The elicitor treatments in virus-inoculated plants showed a significant increase in β–1,3-glucanase activity on the second date of analysis in both tolerant and susceptible varieties. In the inoculated control, the activity was also increased in response to virus infection, but in comparison, in the inoculated treated plants, it was increased tremendously. The β–1,3-glucanase activity was increased in all the treatments on the second date of analysis and then decreased on the third date of analysis even after the third application of elicitor treatments. There was a substantial effect of treatments T2, T3, T6, and T7 on β–1,3 glucanase activity in both tolerant and susceptible varieties on the second date of analysis, and the activity was decreased when treated plants were analyzed on the third date. The maximum β–1,3 glucanase activity increased up to 150 U/mg in SA-treated plants on the second date of analysis, followed by BTH-treated plants. MeJA showed the least effect on β–1,3 glucanase activity, whereas the combined application of SA, MeJA, and BTH also showed increased activity in both the tolerant and susceptible varieties (Figure 9).

#### 3.3.8. Chitinase Activity

**Figure 10:**
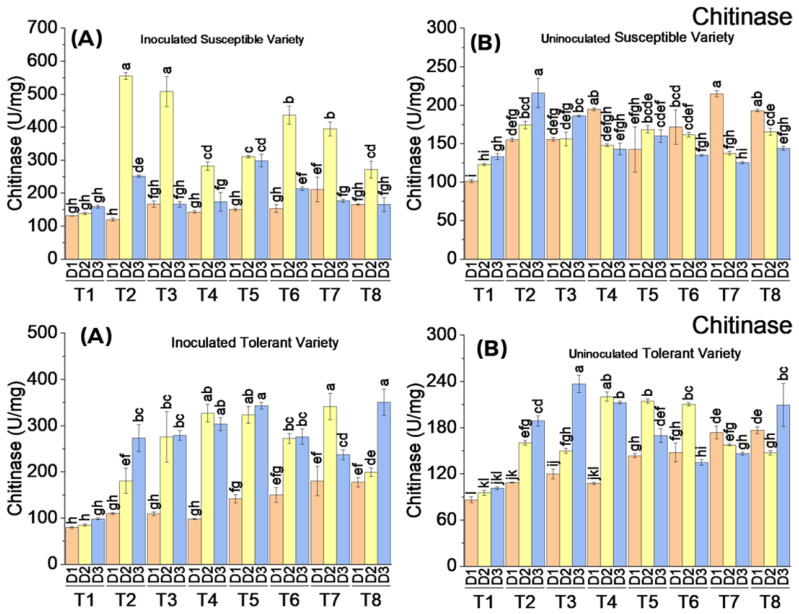
Mean values of chitinase activity in susceptible ((**above A**) inoculated and (**above B**) uninoculated) and tolerant ((**below A**) inoculated and (**below B**) uninoculated) varieties on three dates in response to treatments; T1: control, T2: SA 5 mM, T3: SA10 mM, T4: MeJA1 mM + SA5 mM, T5: MeJA1 mM + SA10 mM, T6: 1.1 mM BTH, T7: 2.2 mM BTH, T8: 1 mM MeJA + 5 mM SA + 1.1 mM BTH. Different letters on bars show significant differences at *p* < 0.05.

All the treatments increased the chitinase activity in virus-inoculated plants, whereas uninoculated treated plants showed a small increase in chitinase activity compared to the control. In the tolerant variety, virus inoculation followed by the application of elicitors resulted in an increase in chitinase activity on the second and third dates of analysis. The maximum chitinase activity was observed to be 351 U/mg on the third date of analysis, where all the elicitors were applied in combination. There was a significant difference in chitinase activity between virus-inoculated and uninoculated plants of the susceptible variety. In the susceptible variety, the chitinase activity was increased in inoculated plants only on the second date of analysis. The maximum increase in chitinase activity was observed to be 554 and 508 U/mg, where cotton plants were treated with SA at concentrations of 5 and 10 mM, respectively. In uninoculated plants, all the treatments showed a low and almost equal quantity of chitinase activity on all dates (Figure 10).

#### 3.3.9. Effect of Different Elicitor Treatments on Overall Enzymatic Activities

The overall comparison of enzymatic activities in response to the exogenous application of different elicitors as treatments on virus-inoculated and uninoculated susceptible and tolerant cotton plants was calculated and analyzed. There was a significant difference (*p* < 0.05) in the treatments for overall enzymatic activities compared to the control. The maximum SOD activity was observed for the treatment of 1 mM MeJA combined with 10 mM SA followed by BTH 1.1 mM treatment. There was a significant effect of treatments on POD activity, and BTH at a concentration of 2.2 mM appeared to be the best treatment for this enzymatic activity. Other treatments, 1 mM SA and 1.1 mM BTH, were also effective for POD activity. Similarly, all the treatments showed a significant effect on CAT activity, and the maximum response was observed where BTH treatments were applied at concentrations of 2.2 and 1.1 mM. The enzymatic activity of PPO showed significantly different responses to the treatments compared to the control. The maximum PPO activity was observed in the response to 1 mM SA application as a treatment. The response of the remaining treatments to PPO activity appeared to be statistically at par. The enzymatic activity of PAL in response to each elicitor treatment was significantly different from that in the control. All the treatments appeared to be statistically significant at par for PAL activity. The production of total phenolic compounds was also significantly affected by all the treatments. The maximum phenolic compounds were produced where the treatment of 1 mM MeJA + 10 mM SA was applied. The second and third most promising responses were observed in the treatments of 1 mM MeJA + 1 mM SA and the combination of 1 mM MeJ A+ 5 mM SA + 1.1 mM BTH, respectively. The response of β–1,3 glucanase activity also appeared to be significant for all the treatments applied compared to the control. Most of the treatments were statistically significant at par for β–1,3 glucanase activity. However, SA applied at a concentration of 1 mM showed a maximum response of β–1,3 glucanase activity. Chitinase activity in response to treatments was significantly different from that in the control. All the treatments appeared to be statistically at par in response to chitinase activity except the treatment of SA at a concentration of 10 mM (Table 1).

### 3.4. Correlation Analysis among the Enzymatic Activities and Disease Severity

**Figure 11:**
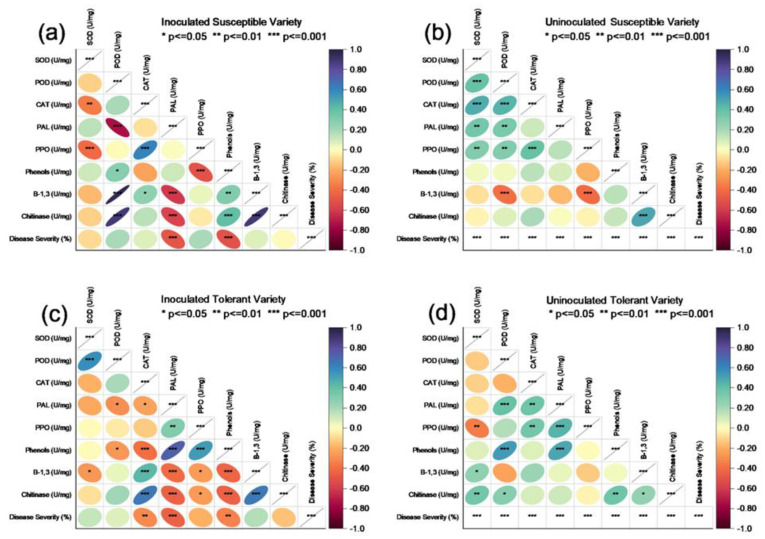
Pearson correlation for different enzymatic activities and disease severity under different elicitors treatments on (**a**,**c**) inoculated and (**b**,**d**) uninoculated susceptible and tolerant varieties.

Correlation analysis revealed a non-significant interaction among most of the enzymatic activities as well as with disease severity in the susceptible cotton variety in response to the elicitor treatments. Only PAL and β–1,3 glucanase enzymatic activities showed a significant relationship with disease severity, with 48 and 40% correlation coefficients, respectively. Disease severity decreased with the decrease in PAL and increased in β–1,3 glucanase activity. There was a highly significant positive correlation of β–1,3 glucanase with chitinase and POD, whereas it showed a highly significant negative correlation with PAL. Similarly, PAL activity was highly correlated with chitinase and POD, PAL increased with a decrease in the activity of chitinase and POD. Contrary to the susceptible cotton variety, most of the enzymatic activities in response to elicitor treatments to suppress CLCuV disease in the tolerant variety showed a significant correlation with disease severity. Only PPO and β–1,3 glucanase activities were not significantly correlated with disease severity. Disease severity showed a highly significant positive correlation with PAL, phenol, and chitinase activity, it increased or decreased with the increase or decrease in enzymatic activities. There was a significant negative correlation between disease severity and POD and CAT, the increased activities later reduced the disease severity. There were variable interactions among different enzymatic activities. PAL activity showed a strong negative correlation with β–1,3 glucanase, chitinase, POD, and SOD, where the enzymatic activities later decreased with the increased activity of PAL. However, PAL showed a positive correlation with phenolic contents. Chitinase activity was positively correlated with POD and SOD, whereas it was negatively correlated with phenol (Figure 11).

### 3.5. Principal Component Analysis

**Figure 12:**
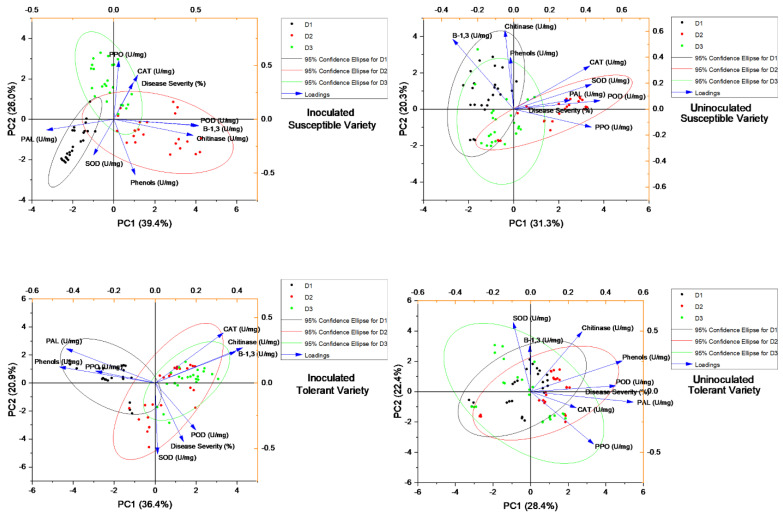
Principal component loading plots and scores of principal component analysis of different treatment applications of elicitors with different concentrations indicates that CAT, β,1−3 glucanase and chitinase have more of a positive impact due to their increased biological activity in the plant for the suppression of the virus. Principal coordinate analysis at the ASV level based on Bray-Curtis distances.

The principal component (PC1) analysis of the uninoculated susceptible variety for different enzymatic, non-enzymatic and PR protein activities at different concentrations of elicitor applications revealed 31.3% (PC1) and 20.3% (PC2) variations, where β−1,3, chitinase and phenols were expressed and grouped significantly at the first date of analysis of elicitor application, while SOD, PAL and PPO were grouped at the second date of analysis of elicitors. No dimension reduction was calculated for the third date of analysis. Similarly, for the inoculated susceptible varieties, 39.4% (PC1) and 26.0% (PC2) variations were calculated wherein only PAL activity was found to be significant for the first date of analysis, POD, β−1,3 and chitinase was reduced for the second date, whereas only PPO was measured for the second date of analysis. Principal component (PC1) analysis of the uninoculated tolerant variety for antioxidants and enzyme activities revealed 28.4% (PC1) and 22.4% (PC2) variations, where only β−1,3, glucanase was dimensionally reduced at the first date of analysis of elicitor application, while chitinase, phenol, POD, PAL and catalase were grouped for the second date of analysis of elicitor application and PPO was only reduced for the third date of analysis. Similarly, for the inoculated tolerant variety, 36.4% (PC1) and 20.9% (PC2) variations were calculated wherein only PAL, phenol and PPO activity was found to be significant for the first date of analysis, while catalase, chitinase, β−1,3, POD and SOD were reduced for the second and third date of analysis after the application of elicitors. The loading plots or arrows or vectors represent the importance or strength of the corresponding variable in the principal component space. Longer arrows indicate that a particular variable has a more significant influence or contributes more to the variation in the data. The relationships among enzymatic, non-enzymatic and PR protein activities at different locations angle of vectors < 90° are positively correlated and vectors with an angle > 90° are not correlated (Figure 12).

## 4. Discussion

Cotton leaf curl virus (CLCuV) disease has been considered among the biggest threats to the cotton industry globally, and particularly in Pakistan for the last three decades. Many strategies have been employed to control this disease, starting from cultural practices, control of its vector whitefly with insecticides, development of resistant varieties by conventional breeding, host, and pathogen-derived resistance, and also with gene silencing. Although these strategies worked, long-term stable control was not achieved [31]. Plants employ a network of signal transduction-dependent and -independent SA pathways that offer great regulatory potential for triggering multiple resistance mechanisms in various combinations [32]. SA is also responsible for the inhibition of viral replication and cell-to-cell movement of viruses by inducing PR proteins through their signals [33,34]. Benzothiadiazide (BTH), a chemical corresponding to SA, is a powerful SAR activator of signal transduction downstream of SA accumulation. BTH has been used effectively against a number of diseases of field crops [35,36,37]. Jasmonic acid (JA) reacts at an early stage of infection and interacts directly with SAR by establishing prompt signals. The plant responds to these signals and activates the defense mechanism to avoid further infection of pathogens [38,39]. SOD, CAT, and POD play vital roles in scavenging excess reactive oxygen species (ROS). These defense-related enzymes are involved in the resistance mechanism of plants [40]. These enzymes are the first line of defense against ROS by protecting cells from oxidative stresses [41].

The PPO and PAL activities were significantly increased in both varieties compared to the control, but these activities were observed to be greater in uninoculated plants than in inoculated plants. The susceptible variety showed more activity than the tolerant variety. The PAL enzyme is involved in the metabolism of phenylpropanoid synthesis, a defense-related secondary metabolite such as phenols and lignin [42,43], and consequently affects the biosynthetic pathways of phenolic compounds [44]. The authors of [45] reported that increased PAL activity is responsible for the suppression of disease severity in resistant tomato cultivars. PPO acts as the first line of defense by catalyzing the oxidation of phenolics to free radicals that can react and create unfavorable conditions for pathogens [46,47]. It seems that both enzymatic activities are interlinked with the production of phenolic compounds. The increase in phenolic compounds in the inoculated susceptible variety suppressed the activity of PAL and PPO, whereas in uninoculated susceptible plants, PAL and PPO activities suppressed phenolic compounds. This would seem to be due to signal transduction occurring more quickly during infection in a susceptible variety. The phenolic contents were observed to be higher in the susceptible variety. Virus inoculation followed by the application of elicitors depicted a distinguished increase in the total phenolic contents of the susceptible variety. Phenolic compounds provide mechanical strength to host cell walls by producing suberine and lignin, which act as a physical barrier to block pathogen entry into the host [48,49]. The increase in total phenolic compounds is mostly found to be higher in resistant genotypes than in susceptible genotypes [50], but our study revealed that the increased level of phenolic content correlated with increased resistance to virus infection more in susceptible varieties when treated with elicitors. The authors of the reference [51] demonstrated a change in disease susceptibility by altering the level of phenolic compounds.

The authors of the reference [52] compared the different enzymatic activities in resistant and susceptible cotton genotypes infected with *Cotton leaf curl Burewala virus* (CLCuBuV) and established the role of SOD, POD, CAT, PPO, PAL, and phenolic compounds in disease resistance. Total phenolic contents and SOD increased significantly in resistant varieties, whereas POD, PPO, and PAL increased non-significantly in both varieties. CAT activity was higher in the susceptible variety than in the resistant variety [52]. Our results are contrary to this study because we used tolerant and susceptible varieties from the same genotype of cotton *Gossipium hirsutum* whereas they used two different genotypes from *G. arboreum* and *G. herbaceum* as resistant varieties and *G. hirsutum* as a susceptible variety. Second, we applied exogenous elicitors as treatments. There are variable responses of CAT activity in infected plants reported in different studies. Scientists demonstrated that resistant cultivars of *G. hirsutum* exhibited higher and earlier-induced levels of PAL, CAT, and POD [53]. An impressive increase in POD activity was recorded in tomato plants infected with Tobacco mosaic virus (TMV) followed by treatment with elicitors [54]. Act12, as an inducer, increased POD activity in tomato plants against *Yellow leaf curl virus* (YLCV) [12]. The authors of [55] found that CAT activity decreased when the plants were infected with *Urdbean leaf crinkle virus* (ULCV). CAT activity in TMV-infected tobacco leaves remained unchanged, whereas decreased CAT activity was observed in cucumber plants infected with CMV [56]. In contrast, CAT activity was significantly increased in Phaseolus vulgaris infected with white clover mosaic virus WClMV [57]. Furthermore, these enzymatic activities have different expression levels in different plants after the onset of infection [52].

Our results revealed that the CLCuMuV-inoculated cotton plants followed by the exogenous application of elicitors, viz., S.A, MeJA, and BTH at different concentrations alone and in combination significantly affected almost all the enzymatic activities, which resulted in the suppression of disease severity. BTH application at both concentrations, 1.1 mM and 2.2 mM, appeared to be the most effective treatment regarding the overall enzymatic activities of SOD, POD, and CAT. The application of 1 mM MeJA combined with 10 mM SA proved to be the best for the enhanced production of SOD, PAL, and total phenolic contents. The application of 1 mM SA appeared to be the most effective treatment for increasing the enzymatic activity of PPO and β–1,3 glucanase, whereas 10 mM SA treatment showed more enzymatic activity of chitinase compared to other treatments. While the exogenous application of elicitors significantly increased the enzymatic activities, it also significantly enhanced the resistance in cotton plants against CLCuMuB compared to the inoculated control. In a tolerant variety, the disease was suppressed in all the treatments. BTH treatment at a concentration of 1.1 mM proved to be the best treatment with zero percent disease severity. However, the 2.2 mM BTH treatment in the tolerant cotton variety showed only 1.23% disease severity. In susceptible cotton varieties, virus infection was suppressed by the application of treatments compared to the control. Similar to the tolerant variety, the most effective BTH treatment was applied at both concentrations. The disease severity remained at 3.39% and 3.53% with the treatments of 1.1 mM and 2.2 mM BTH, respectively. The viral coat protein region was amplified by PCR from all the treated tolerant cotton plants except BTH, which was applied at a concentration of 1.1 mM. However, all the treated plants of the susceptible variety gave positive PCR amplification with the AV/AC core primer for viral coat protein. The beta satellite component of CLCuMuVB involved in the formation of leaf enation was also detected by PCR from the treated cotton tolerant and susceptible varieties. In a tolerant variety, the beta satellite was not amplified from plants treated with BTH alone at either concentration or in combination with MeJA and SA. However, in the susceptible variety, only BTH-treated plants at both concentrations did not show any amplification. Our results reflect that although the virus was present in most of the treated plants, the beta component was absent in BTH-treated plants. This shows that there are certain PR proteins, e.g., β–1,3 glucanase, chitinase, PR1 and PR2 produced in the response of SAR that interact with viral proteins that hinder the replication of beta satellite components. BTH proved to be the best treatment to induce SAR, which ultimately suppressed virus multiplication. The response of the tolerant variety against the virus may be due to its inherent ability to combat more efficiently, which was effectively enhanced by BTH treatment.

Our results are in line with previous work on disease suppression by the application of different signaling compounds, such as SA, MeJA, and BTH. Different studies strongly support the significant role of SA in the suppression of RNA viruses, whereas there are no appreciable results regarding the suppression of DNA viruses. The authors of [58] studied the resistance mechanism induced by SA in Ring Red pear and its effect on the severity of disease and plant defense enzymes. Interestingly, it was possible to effectively increase the resistance of pears to *P. piricola* through treatment with 0.2 mM SA. SA treatment improved PPO, PAL, POD, chitinase, and β−1,3-glucanase production in the inoculated leaves. PR-proteins were found to respond quickly to microbes. These findings showed that SA could provide systemic acquired resistance and reinforce pear’s antimicrobial ability to *P. piricola*. RNA viruses activate the SA pathway [59]. Virus resistance was triggered by exogenous application of SA, which caused a reduction in viral load and delay in the appearance of symptoms of the disease [60,61]. The authors of the reference [62] elucidated the role of exogenous application of SA in increasing the resistance of Phaseolus vulgaris against tobacco necrosis virus (TNV). Two-week-old plants with primary bean leaves treated with 0.1, 0.5, and 1-mM SA showed fractional inhibition of the growth of local lesions. This inhibition also increased POD activity. Such information has recently been revealed for geminivirus infections [62].

The defense reactions in potato were stimulated by the increased activities of both chitinase and β–1,3-glucanase against early and late blight diseases by the application of elicitors [63]. In the same way, resistance was observed in tobacco and Arabidopsis by the elevated levels of β–1,3-glucanase and chitinase [64]. An isoform of chitinase PRQ was induced by TMV into the extracellular spaces and decreased its infection in tobacco [65]. Higher chitinase activity was found in the tomato plants inoculated with TMV followed by the elicitor treatments [54]. Β–1,3-glucanase, in association with some pathogenesis-related enzymes, is responsible for the stimulation of defensive responses and decreases the disease incidence against rhizome rot in turmeric [66]. In our study, β–1,3 glucanase activity was significantly increased in virus-inoculated plants following the application of elicitors, and this activity remained higher in the susceptible variety. The β–1,3-glucanase activity was increased only on the second date of analysis and then decreased on the third date of analysis. Our findings are in line with [67], who observed that β–1,3-glucanase activity either decreased or remained the same in resistant and moderately resistant cotton genotypes. Similarly, there was a significant increase in chitinase activity in virus-inoculated plants, but there was a non-significant difference in susceptible and tolerant varieties, although it increased in both. Chitinase activity was elevated on the second date of analysis and decreased on the third date of analysis. Whereas, our findings are contrary to the study of [68], who demonstrated that rapid and enhanced activities of β–1,3-glucanase and chitinase correspond to higher levels of resistance in cotton.

The responses of plants against different pathogens vary in terms of how plants react to a specific pathogen. Normally, when a pathogen attacks plants, a hypersensitive response occurs at the onset of infection because of early signaling. These signals halt the further invasion of the pathogen by activating SAR, which confers plants with different eliciting molecules. In our study, most enzymatic activities were higher in the susceptible variety than in the tolerant variety, which might be due to vigorous signaling in response to a compatible reaction of the virus and susceptible cotton variety. Furthermore, the exogenous application of elicitor (signaling molecule) treatment triggered SAR more rapidly, and thus, enzymatic activities were increased. A global study of the transcriptome of Arabidopsis in infectious cabbage hosts caused by cabbage leaf curl virus (CaLCuV), which belongs to the geminivirus, was conducted by [69]. CaLCuV elicits the microbe response through the SA pathway. In addition, they also found that plants where SA was elicited were resistant to CLCuV infection, suggesting that this SA-mediated pathway significantly reduced the infection of geminivirus. The authors of the reference [49] revealed that SA enhances a cascade of events resulting in the inhibition of viral multiplication and their cell-to-cell and long-distance spread in plants [70]. The exogenous application of SA was shown to mimic certain characteristics of an infection resulting in the induction of resistance [33,71]. The SA pathway has been reported to be activated by RNA viruses [59]. The foliar application of SA is generally reported to induce resistance to viruses, which is characterized by a decrease in virus titer and a delay in the establishment of disease symptoms [61,72].

The authors of the reference [73] revealed that jasmonic acid and SA have their defense pathways. These defense pathways work simultaneously to provide plants with regulatory potential. This potential enables plants to defend themselves against any type of pathogen [73,74,75,76]. Foliar application increases the resistance of plants to ZYMV and plays a significant role in triggering the induced protection response against ZYMV [77]. Resistance to various viral diseases, such as TMV, TCV, and CMV, was induced and improved in Arabidopsis, tobacco, tomato, and hot pepper. This tolerance was elevated because of the exogenous application of 0.06 mM JA followed by 0.1 mM SA [34,35]. The concentration of viral DNA showed a 5–6-fold decrease in BTH-treated plants; thus, virus replication was heavily affected in BTH-treated plants [78]. Resistance against powdery mildew infection is increased in strawberry leaves by the deposition of soluble phenolic compounds, which are enhanced using BTH [79]. If BTH is applied at higher concentrations in plants, it leads to the continuous activation of immune responses in plants, including some protective genes [80,81]. In relatively low dosages of BTH, the protective response by plants is not automatically triggered but only becomes visible following infection [82].

## 5. Conclusions

The exogenous application of BTH could be helpful in enhancing the resistance of cotton plants against CLCuMuV by enhancing the SAR mechanism after its induction through virus infection. We obtained satisfactory results of BTH application as an induced resistance enhancer, which has established some facts about its exogenous application and the suppression of CLCuMuV in cotton plants. It was previously established that the SAR mechanism works better and significantly suppresses the RNA viruses as a result of elicitor application, but there were no significant results on DNA begomovirus. It was revealed that in response to virus infection, and after the onset of SAR, the application of BTH as a signaling compound may further enhance some specific PR proteins, e.g., β–1,3 glucanase, chitinase that interact with betasellite of CLCuMuV, which is considered for symptom induction. Therefore, symptoms were not expressed. It is imperative to study the gene expression of PR proteins, e.g., β–1,3 glucanase, chitinase, PR1 or PR2 and their interactions with viral proteins and the effect of BTH under field conditions. After obtaining satisfactory results from the abovementioned future studies, the BTH application would be part of the CLCuV integrated disease management program.

## Figures and Tables

**Table 1:** Comparison of different elicitor treatments on enzymatic activities under glasshouse conditions.

	Enzymatic Activities ± SE							
Treatments	SOD	POD	CAT	PPO	PAL	Phenols	β−1,3 Glucanase	Chitinase
Control	451.94 d ± 50.90	224.74 f ± 6.57	360 e ± 48.39	5.66 e ± 0.74	66 e ± 3.18	50.06 g ± 3.82	21.78 d ± 1.13	175.09 c ± 9.35
S.A 1 mM *	795.07 b ± 65.48	402.46 b ± 12.64	708.9 d ± 31.01	11.22 a ± 0.83	162 c ± 2.65	109.50 c ± 2.87	44.53 a ± 2.96	207.68 b ± 9.39
S.A 10 Mm	718.30 bcd ± 96.87	295.90 e ± 8.80	767.75 c ± 51.70	7.75 d ± 0.80	158 d ± 4.95	97.55 e ± 4.80	37.46 b ± 2.29	227.97 a ± 14.17
MeJA 1 mM + S.A 1 mM	630 cd ± 34.21	311.26 d ± 8.29	707.51 d ± 46.95	9.25 b ± 0.24	165 b ± 2.96	114.22 b ± 3.09	37.57 b ± 3.40	203.13 b ± 8.58
MeJA 1 mM + S.A 10 mM	1115.4 a ± 54.61	339.45 c ± 14.10	894.75 b ± 37.34	9.49 b ± 0.51	169 a ± 3.40	135.97 a ± 4.62	34.95 bc ± 3.39	213.95 b ± 10.25
BTH 1.1 mM	818.92 b ± 64.17	393.92 b ± 15.26	970.7 a ± 59.32	8.42 c ± 0.24	165 b ± 3.79	87.56 f ± 3.64	35.77 bc ± 2.78	205.25 b ± 10.93
BTH 2.2 mM	803.86 b ± 37.99	447.96 a ± 10.11	982 a ± 39.65	9.66 b ± 0.27	166 ab ± 2.86	104.70 d ± 3.99	37.53 b ± 2.47	208.21 b ± 12.78
MeJA 1 mM+ S.A 5 mM + 1.1 mM BTH	758.03 bc ± 31.16	294.92 e ± 8.23	886.53 b ± 34.90	9.42 b ± 0.28	166 ab ± 4.78	111.12 bc ± 5.20	33.12 c ± 2.19	205.93 b ± 10.60
LSD *	159.06	14.05	50.47	0.53	3.44	4.65	2.83	12.31

Different letters show significant differences between groups at *p* < 0.05; S.E.: standard error; mM: milli mole; SOD = superoxide dismutase; POD = peroxidase; CAT = catalase; PPO = polyphenol oxidase; PAL = phenylalanine ammonia-lyase; * LSD = least-significant difference.

## Data Availability

The data presented in this study are available on request from the corresponding author. The data are not publicly available due to privacy.
